# Follicular Adenomas Exhibit a Unique Metabolic Profile. ^1^H NMR Studies of Thyroid Lesions

**DOI:** 10.1371/journal.pone.0084637

**Published:** 2013-12-23

**Authors:** Stanisław Deja, Tomasz Dawiskiba, Waldemar Balcerzak, Magdalena Orczyk-Pawiłowicz, Mateusz Głód, Dorota Pawełka, Piotr Młynarz

**Affiliations:** 1 Faculty of Chemistry, Opole University, Opole, Poland; 2 Department of Vascular, General and Transplantation Surgery, Wrocław Medical University, Wrocław, Poland; 3 First Department and Clinic of General, Gastroenterological and Endocrinological Surgery, Wroclaw Medical University, Wrocław, Poland; 4 Department of Chemistry and Immunochemistry, Wroclaw Medical University, Wrocław, Poland; 5 Department of Bioorganic Chemistry Wrocław University of Technology, Wrocław, Poland; Imperial College London, United Kingdom

## Abstract

Thyroid cancer is the most common endocrine malignancy. However, more than 90% of thyroid nodules are benign. It remains unclear whether thyroid carcinoma arises from preexisting benign nodules. Metabolomics can provide valuable and comprehensive information about low molecular weight compounds present in living systems and further our understanding of the biology regulating pathological processes. Herein, we applied ^1^H NMR-based metabolic profiling to identify the metabolites present in aqueous tissue extracts of healthy thyroid tissue (H), non-neoplastic nodules (NN), follicular adenomas (FA) and malignant thyroid cancer (TC) as an alternative way of investigating cancer lesions. Multivariate statistical methods provided clear discrimination not only between healthy thyroid tissue and pathological thyroid tissue but also between different types of thyroid lesions. Potential biomarkers common to all thyroid lesions were identified, namely, alanine, methionine, acetone, glutamate, glycine, lactate, tyrosine, phenylalanine and hypoxanthine. Metabolic changes in thyroid cancer were mainly related to osmotic regulators (taurine and *scyllo*- and *myo*-inositol), citrate, and amino acids supplying the TCA cycle. Thyroid follicular adenomas were found to display metabolic features of benign non-neoplastic nodules and simultaneously displayed a partial metabolic profile associated with malignancy. This finding allows the discrimination of follicular adenomas from benign non-neoplastic nodules and thyroid cancer with similar accuracy. Moreover, the presented data indicate that follicular adenoma could be an individual stage of thyroid cancer development.

## Introduction

 Thyroid nodules are extremely common. It is estimated that in the general population they are found in 4-7% by palpation and in 50-67% by ultrasonography or at autopsy [1–3]. Although more than 90% of thyroid nodules are benign (mostly hyperplastic, colloid or cystic nodules and less frequently, inflammatory nodules or thyroid adenomas), the possibility of malignancy should always be taken into consideration [2,4]. Thyroid cancer is the most common endocrine malignancy, accounting for 90% of all neuroendocrine malignancies and 1% of all malignant diseases [5]. The worldwide incidence of thyroid carcinoma is variable, with 19.7 new cases per 100,000 in North America, 8.3 per 100,000 in Central Europe, and 3.5 cases per 100,000 in areas of Southern Africa [6]. These carcinomas occur three times more often in females and are more common in younger patients [7]. More than 95% of thyroid cancers originate from thyroid follicular epithelial cells and include well-differentiated papillary carcinomas (75-80%) and follicular carcinomas (10-20%) in addition to poorly differentiated and anaplastic carcinomas (1-2%) [2,7]. Approximately 3% to 5% of all thyroid cancers are parafollicular C-cell derived medullary thyroid carcinomas [2,7]. The other primary proliferative diseases of the thyroid, such as lymphoma and sarcoma, are extremely rare, as are metastatic tumors localized in the thyroid gland. 

 The most important diagnostic tool for thyroid nodules is the cytological examination of the samples obtained by fine-needle aspiration biopsy (FNAB) [1,5,8,9]. Unfortunately, retrospective studies of FNAB diagnosis show the limitations of this tool; the nodules with associated indeterminate or suspicious cytology represent 9-24% of cases. Furthermore, approximately 15% of all biopsies are assessed as insufficient for diagnosis [2,9]. Immunocytochemical analyses and RT-PCR techniques are applied to increase the diagnostic accuracy of FNAB. Useful markers of thyroid tumors include the following: galectin-3, E-cadherin, fibronectin, CD44v6, thyroid transcription factor 1, Cbp/p300-interacting transactivator 1, thyroglobulin, calcitonin, CEA, p27, cyclin D1, cytokeratin 19, thyroid peroxidase, HBME-1, beta-catenin, and p53 [7–11]. 

 Metabolomics is defined as the comprehensive and simultaneous profiling of metabolites within an organism, cell, or tissue [12]. Therefore, metabolomics is the most downstream approach among all “omics” approaches. An observation of changes at the level of metabolites has a substantial advantage in comparison to gene, transcript or protein analysis, because it is more rapid in terms of its detection of perturbations occurring in metabolic networks. There are several thousand metabolites in cells and organisms; most of these metabolites are known, and their functions have been well-defined. These metabolites are an important source of information about living systems. Despite the enormous number of metabolites, relatively few of these compounds are defined as biomarkers that exhibit changes related to different physiological states and describe the dynamic changes caused by a disease or external stimuli [13].

 As previously shown, Nuclear Magnetic Resonance (NMR)-based metabolomics is a powerful tool for the quantitative and qualitative analysis of metabolites present in tissues and various biofluids, such as urine, serum, cerebrospinal fluid and saliva [14–16]. NMR spectra reflect the content of low molecular weight compounds (MW < 1500 Da) in the analyzed material. They constitute highly informative metabolic profiles, which can be used as an input for discriminant analysis.

 There are many examples of the successful application of this approach in the field of cancer diagnostics, including in the diagnosis of kidney [17], lung [18], breast [19], prostate [20], colon [21] and other tumors. However, the metabolomics platform has been applied only a few times in thyroid cancer studies [22,23]. The first studies, conducted by Mountford et al., demonstrated the potential of ^1^H NMR to differentiate between malignant and healthy postoperative thyroid tissue specimens, but the results obtained were ambiguous. According to their criteria for the investigation of follicular thyroid lesions, 60% of adenomas were similar to malignant lesions, while the remaining 40% were similar to normal thyroid tissues [24–26]. The authors suspected that a group of follicular lesions might progress through early malignant changes as was previously reported for adenoma-carcinoma in colonic neoplasms [27,28]. Most recently, the potential of HR MAS spectroscopy measurements to achieve highly accurate diagnosis in thyroid tumor fine-needle aspiration biopsies has been reported in the literature [29,30]. This approach enables discrimination not only between normal and malignant tissues but also between malignant and benign lesions. 

 While most of the metabolomics studies have focused on diagnostic applications that lead to a separation between control and cancer subject groups and to the development of biomarkers, there still have been no attempts made towards explaining the potential molecular mechanisms of thyroid cancer. The metabolomics approach may provide valuable information in the field of thyroid cancer biology and will be useful in improving our understanding of this disease and adjusting the treatment and monitoring of patients. In the present study, ^1^H NMR-based metabolic profiling combined with multivariate data analysis tools have been applied to investigate the alterations in metabolites present in aqueous tissue extracts (ATE) obtained from healthy thyroid (H), non-neoplastic nodules (NN), follicular adenomas (FA) and thyroid cancer (TC) lesions. The differences in the metabolic profiles allowed for the identification of the most important metabolites associated with different thyroid lesion progression. The relevant disease-related metabolic pathways are discussed here, and FA is suggested to be an intermediate step in carcinogenesis.

## Materials and Methods

### Clinical population and sample collection

 Tissue samples were collected from patients who were operated on at the First Department and Clinic of General, Gastroenterological and Endocrinological Surgery of Wrocław Medical University. The protocol for this study was approved by the Commission of Bioethics at the Wrocław Medical University (Approval no. KB-248/2010), and written informed consent was obtained from all the patients before enrollment in the study. All patients were euthyroid and had a normal level of thyroid-stimulating hormone (TSH). They were not treated with any drugs before the surgery. Thyroid specimens (the tumor tissues and the healthy tissues from the opposite thyroid lobe that is routinely resected in such situations) were collected intraoperatively. Each sample was snap frozen in liquid nitrogen and stored at -80°C. Histological assessment and classification were conducted according to the criteria of the WHO [31]. 

 The cases of hyperplastic, colloid and cystic nodules were recognized jointly as non-neoplastic nodules. The differences between hyperplastic and colloid nodules are related mainly to the amount of colloid and to cellularity. True thyroid cysts are rather rare, and most of the so-called cystic nodules are “pseudocysts” created as a result of nodular colliquation necrosis [4]. The neoplastic nodules were divided into benign follicular adenomas and cases of thyroid carcinomas.

 A cohort of 31 patients with histopathologically confirmed malignant and benign thyroid tumors was examined and provided 64 tissue samples comprising 45 lesion and 19 healthy thyroid samples (details in Table 1). All patients were Caucasian.

**Table 1:** Summary of the tissue specimens collected from the patients.

**Group**	**No. of patients**	**Age**	**No. of tissue specimens**
	**n (male/female)**	**mean ± SD**	**n (male/female)**
**Lesions**	---	---	---
non-neoplastic nodules - NN	10 (10/0)	61.7 ± 8.1	16 (16/0)
follicular adenomas - FA	9 (7/2)	40.1 ± 11.7	14 (9/5)
thyroid carcinomas - TC	12 (10/2)	57.5 ± 17.7	15 (12/3)
**Total**	31 (27/4)	53.6 ± 15.8	45 (37/8)
**Healthy**	19 (17/2)	52.5 ± 14.5	19 (17/2)

### Sample handling and NMR spectroscopy

 Prior to NMR measurements, thyroid tissue fragments were weighed and frozen in liquid nitrogen for at least 10 minutes. The tissues were then transferred into a mechanic steel bead homogenizer (Tissuelyser LT; QIAGEN, Hilden, Germany). During the first step of homogenization, frozen tissue samples were disrupted into powder for 10 minutes at 50 Hz shaking frequency. The obtained powder was suspended in 1 mL of 0.5 M phosphate buffer solution (PBS), (pH=7.4; 10% D_2_O; 3 mM sodium azide; 1 mM trimethylsilyl-2,2,3,3-tetradeuteropropionic acid sodium salt (TSP) as an internal standard). Next, homogenization was performed again for 10 minutes with the same shaking frequency. The solutions were placed in an ultrasound bath and sonicated for 10 minutes. To remove any cellular debris or insoluble material, samples were centrifuged at 15 000 x g for 20 minutes. Finally, 500 μL aliquots of supernatant of aqueous tissue extract (ATE) were transferred into 5 mm NMR tubes and stored in 4°C until measurements. 

 All NMR spectra were recorded at 300 K with a 600 MHz NMR spectrometer (Bruker Biospin Avance II; Bruker, GmBH, Germany) operating at a proton frequency of 600.58 MHz. The extraction of metabolites using aqueous buffer solution results in high protein content of the sample. Therefore, a one-dimensional Carr-Purcell-Meiboom-Gill (CPMG) spectrum with water presaturation was acquired to filter out any broad resonances arising from the presence of proteins and to enable observation of low molecular weight compounds (number of loops = 80, spin echo delay = 400 μs). Each spectrum consisted of 128 scans and was stored in 64k data points. After Fourier transformation of spectra, the baseline and phase were manually corrected using Topspin 1.3 software (Bruker, GmBH, Germany). Signal assignments were performed based on the in-house and online (HMDB, BMRB) databases and confirmed using two-dimensional NMR experiments: ^1^H-^1^H COSY, ^1^H-^1^H TOCSY and ^1^H-^13^C HSQC.

### Data analysis: chemometrics and statistics

 A total of 64 spectra of ATE corresponding to 45 thyroid lesions and 19 healthy thyroid tissues were considered for multivariate analysis. The preparation of ATE using PBS results in a highly reproducible ^1^H NMR spectra. The whole spectral set was shifted to the lactate methyl signal (δ = 1.325 ppm) and alignment based on the icoshift algorithm [32] was performed only for most shifted regions e.g., citrate (region 2.48 - 2.68 ppm). The data consisted of 25767 points (region 0.5 - 10.0 ppm) and after exclusion of the residual water signal region (4.53 - 5.15 ppm), resulted in 23999 variables (X matrix). A multivariate data analysis was performed using the SIMCA-P+ software (v 13.0, Umetrics, Umeå, Sweden). The data were normalized using a probabilistic quotient (PQ) normalization method [33] and scaled using Pareto scaling. Principal Component Analysis (PCA) was utilized for data overview and outlier detection. A Y table was created based on postoperative histopathological diagnosis; this served to define classes in Orthogonal Projections to Latent Structures Discriminant Analysis (OPLS-DA) modeling [34], and the default 7-fold cross-validation was applied (1/7 of the samples being excluded from calculations in each round). To provide evidence that the observed separation is not due to random effects, all models were validated by cross validation analysis of variance (CV-ANOVA) at a level of significance: α = 0.05. 

 The STATISTICA software (v 10, StatSoft, Tulsa, USA) was utilized for the statistical analysis of the quantified metabolites (in terms of signal integrals). In this case, only identified signals without overlap were used. In a univariate analysis, a statistical importance was tested with Kruskal-Wallis one-way ANOVA (KW ANOVA), Student's t-test and Mann-Whitney-Wilcoxon (MWW) test. For nonparametric MWW test and KW ANOVA signal integrals were used, while Student's t-test was performed on logarithm transformed data to approximate normal distribution [35].

 To identify the most changed metabolic pathways in thyroid lesions, a set of significantly affected metabolites were used as an input for the Metabolite Set Enrichment Analysis (MSEA) [36]. The MSEA is a freely available online tool that can be found at (http://www.msea.ca/MSEA/faces/Home.jsp). The MSEA is an extension of Gene Set Enrichment Analysis. Over Representation Analysis (ORA) was utilized for comprehensive screening of affected pathways. After adjusting for multiple-testing, fold enrichment and one-tailed *P*-values are reported

## Results

### Metabolic profile of thyroid tissue homogenates

 Metabolic profiles of thyroid aqueous tissue extracts exhibit a variety of metabolites belonging to few biochemical groups: amino acids, organic acids, lipids and carbohydrates. A total of 28 metabolites were identified (Table 2). To obtain an overview of the dataset, a multivariate data analysis approach was applied. 

**Table 2:** Identified metabolites in ^1^H NMR spectra of aqueous thyroid tissue extracts.

**No**	**Metabolite**	**H group**	δ 1H NMR (multiplicity*)
1	Isoleucine	α-CH; β-CH; β-CH_3_; γ-CH_2_(i); γ-CH_2_(ii); δ-CH_3_	3.66 (d); 1.99 (m); 1.01 (d); 1.27 (m); 1.48 (m); 0.95 (t)
2	Valine	α-CH; β-CH; γ-CH_3_; γ'-CH_3_	3.62 (d); 2.27 (m); 1.00 (d); 1.04 (d)
3	3-Hydroxybutyrate	γ-CH_3_	1.19 (d)
4	Alanine	CH; CH_3_	3.78 (q); 1.49 (d)
5	Acetate	CH_3_	1.93 (s)
6	NAC	NHCOOH_3_	2.02 (s)
7	Methionine	α-CH; β-CH_2_; γ-CH_2_; S-CH_3_	3.87 (t); 2.15 (m); 2.65 (t); 2.14 (s)
8	Acetone	β-CH_3_	2.22 (s)
9	Glutamate	α-CH; β-CH_2_; γ-CH_2_	3.77 (t); 2.08 (m); 2.36 (m)
10	Succinate	2xCH_2_	2.41 (s)
11	Citrate	CH_2_ (i); CH_2_ (ii)	2.65 (d); 2.51 (d)
12	Creatine	N-CH_3_; CH_2_	3.02 (s); 3.92 (s)
13	Choline	N-(CH_3_)_3_	3.18 (s)
14	PC	N-(CH_3_)_3_	3.19 (s)
15	GPC	N-(CH_3_)_3_	3.20 (s)
16	Scyllo-inositol	6xCH	3.34 (s)
17	Taurine	N-CH_2_; SO_3_-CH_2_	3.43 (t); 3.28 (t)
18	Glycine	CH	3.55 (s)
19	Myo-inositol	H5; H4,H6; H1,H3; H2	3.30 (t); 3.63 (dd); 3.52 (m); 4.08 ()
20	Lactate	β-CH_3_; α-CH	1.33 (d); 4.12 (q)
21	Threonine	α-CH; β-CH; γ-CH_3_	3.59 (d); 4.26 (m); 1.34 (d)
22	β-Glucose	H1; H2; H3; CH2	4.66 (d); 3.25 (dd); 3.48 (t); 3.90 (dd)
23	Tyrosine	2,6-CH; 3,5-CH; α-CH; β-CH_2_	7.20 (d); 6.90 (d); 3.95 (dd); 3.20 (dd), 3.07 (dd)
24	Phenylalanine	2,6-CH; 4-CH; 3,5-CH	7.34 (m); 7.38 (m); 7.43 (m)
25	Uracil	5-CH; 6-CH	5.81 (d); 7.55 (d)
26	Hypoxanthine	8-CH; 2-CH	8.22 (s); 8.20 (s)
27	Formate	CH	8.46 (s)
28	Histidine	4-CH; 2-CH	7.10 (s); 7.89 (s)

^*^ s, singlet; d, doublet; t, triplet; m, multiplet; q, quartet; dd, double doublet; NAC: *N*-acetylated compounds; PC: phosphocholine; GPC: glycerophosphocholine. Underlined signals were used for quantification.

### Discrimination between healthy and pathological thyroid tissues

**Figure 1:**
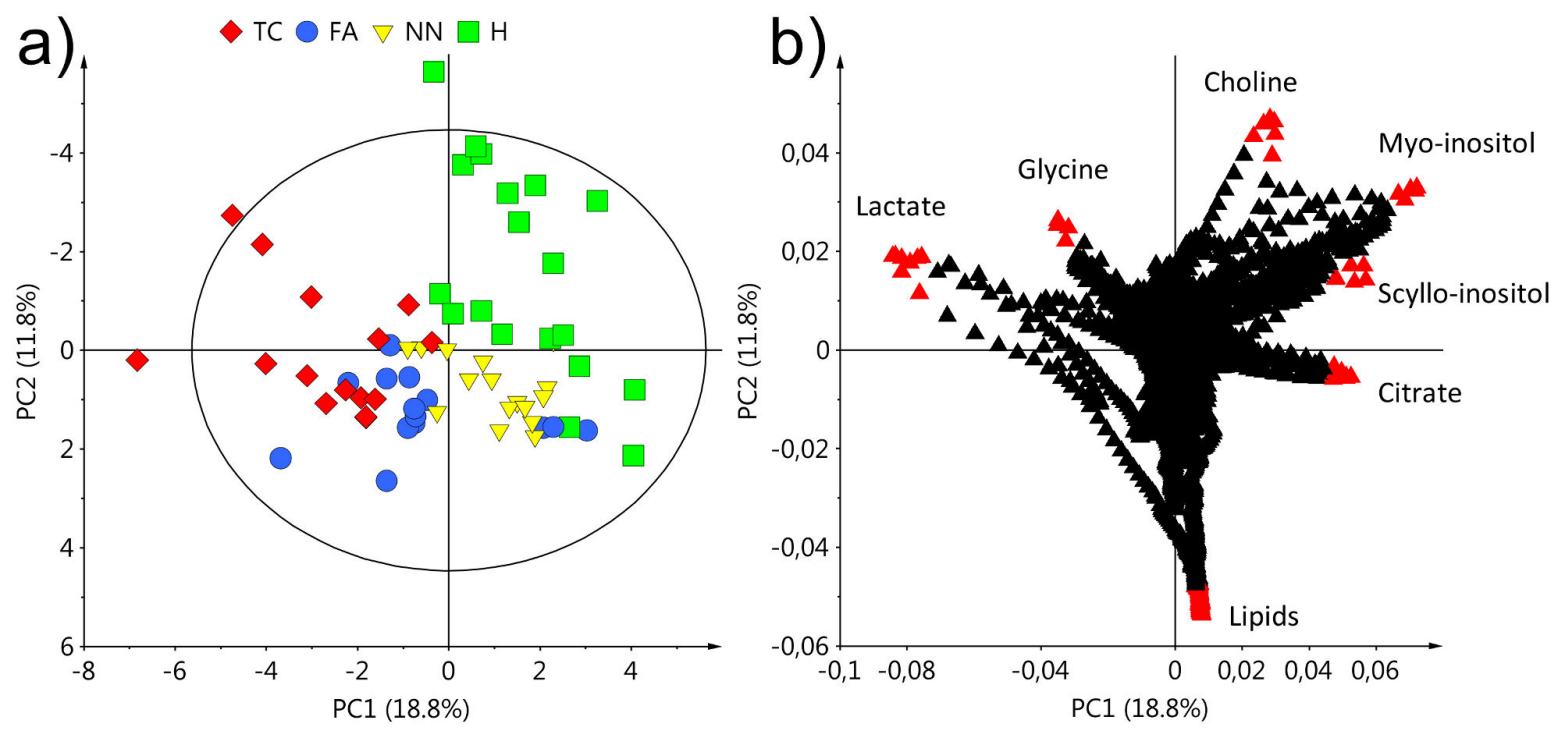
PCA results: (a) score plot and (b) corresponding loading plot based on all ^1^H NMR spectra of ATE. Healthy thyroid - H, non-neoplastic nodules - NN, follicular adenomas - FA, thyroid cancer - TC.

^1^H CPMG NMR spectra of ATE were subjected to multivariate data analysis involving an unsupervised method (PCA), and supervised modeling (OPLS-DA). Initially, a PCA analysis was performed for healthy and all thyroid lesions groups (NN, FA, TC) with two principal components, *R* ^*2*^ *X* = 0.388 and, *Q* ^2^ *Y* = 0.238. The obtained score plot (Figure 1a) shows a clear separation between healthy ATE and all pathological classes. Interestingly, in the PCA score plot, FA lesions were not as homogeneous a group as the other groups. Three FA samples of the total fourteen behaved similarly to healthy thyroid. However, the majority of FA samples formed a separate group between TC and NN. Based only on the unsupervised technique, all thyroid lesion types were well discriminated from healthy thyroid tissues by the following metabolites: lactate, glycine, choline, *myo*-inositol, scyllo-inositol, citrate and lipids (Figure 1b). 

**Figure 2:**
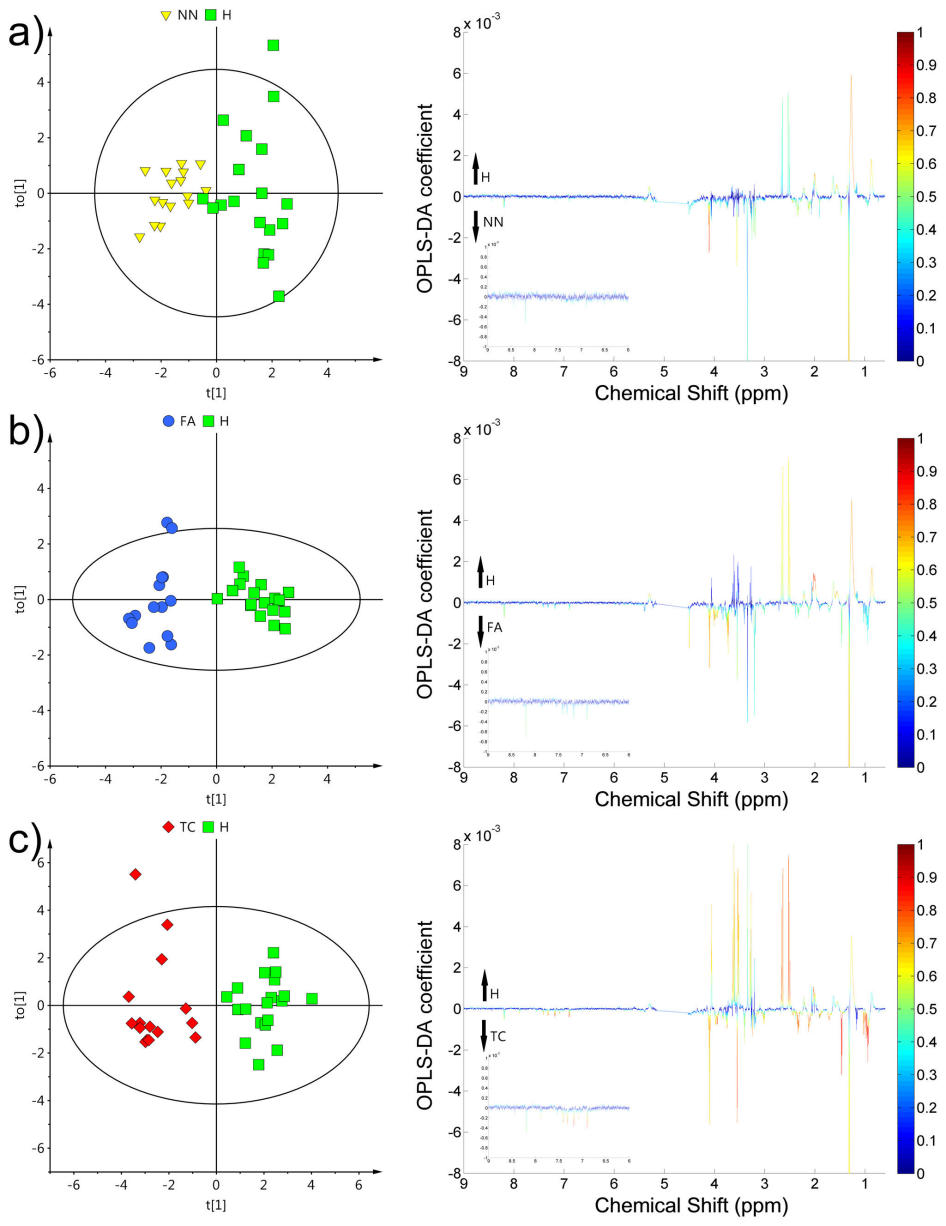
OPLS-DA results and corresponding loadings of discrimination between healthy ATE and different pathological ATE: (a) non-neoplastic nodules - NN, (b) follicular adenomas - FA, (c) thyroid cancer - TC. The color bar corresponds to the absolute value of the correlation loading in the discrimination model.

 The healthy ATE were subsequently compared individually with different pathological ATE resulting in three supervised OPLS-DA models: NN vs. H, FA vs. H and TC vs. H and the separation obtained was better than in PCA (Figure 2). A satisfactory separation of healthy ATE from most benign thyroid lesion (NN) was obtained with one predictive and one orthogonal component (*R* ^*2*^ *X* = 0.325, *R* ^*2*^ *Y* = 0.778 *Q* ^2^ *Y* = 0.549) (Figure 2a). In comparison to PCA, when OPLS-DA was preformed, the FA group became compact and very well distinguished from healthy ATE (FA vs. H), with one predictive and one orthogonal component (*R* ^*2*^ *X* = 0.247, *R* ^*2*^ *Y* = 0.896, *Q* ^2^ *Y* = 0.602) (Figure 2b). Finally, the best separation was achieved in the TC vs. H comparison, with one predictive and one orthogonal component (*R* ^*2*^ *X* = 0.376, *R* ^*2*^ *Y* = 0.881, *Q* ^2^ *Y* = 0.782) (Figure 2c). In summary, all models resulted in predictive values of *Q* ^*2*^ *Y* and a satisfactory separation of each thyroid lesion from H was obtained. All models were statistically significant, with a *P*-value < 0.0001 after testing with CV-ANOVA.

 In addition to the chemometric analysis, a univariate statistical analysis was also performed. The identified metabolites that were statistically significant for at least one comparison are listed in Table 3. Nine metabolites were found to be significantly different in both statistical tests for all three thyroid lesion groups when compared to healthy thyroid tissue. The common biomarkers of pathological thyroid tissue were present at elevated levels and included: methionine, alanine, glutamate, glycine, lactate, tyrosine, phenylalanine and hypoxanthine, while only acetone was at a significantly lower level in tumors than in healthy tissues (Table 3).

**Table 3:** Statistically important metabolites contributing to the subsequent separation of thyroid lesions from healthy thyroid tissue.

**Metabolite**	**% difference**			**% RSD**			
	**NN vs. H**	**FA vs. H**	**TC vs. H**	**H**	**NN**	**FA**	**TC**
Isoleucine	4.3	76.7^a,b^	73.8^a,b^	27.6	19.7	96.8	34.9
Valine	-1.9	83.8^a,b^	104.8^a,b^	31.0	14.8	90.2	35.6
3-Hydroxybutyrate	-20.4^a,b^	-32.2^a,b^	5.9	31.5	27.9	55.5	86.5
Alanine	19.1^a,b^	81.7^a,b^	126.6^a,b^	21.9	23.6	56.4	28.7
Acetate	23.0^a^	16.1	27.9^a,b^	26.5	28.6	26.8	21.9
NAC	-7.7^b^	-38.6^a,b^	-34.3^a,b^	16.7	33.4	17.8	19.9
Methionine	32.4^a,b^	37.6^a,b^	82.5^a,b^	24.4	24.4	25.4	23.0
Acetone	-30.2^a,b^	-36.5^a,b^	-39.2^a,b^	38.3	74.2	53.5	30.6
Glutamate	53.4^a,b^	57.6^a,b^	103.9^a,b^	29.1	54.5	43.3	40.4
Succinate	39.7^a,b^	71.5	63.0^a,b^	33.5	42.4	68.4	37.4
Citrate	-24.8	-66.5^a,b^	-85.4^a,b^	48.1	41.7	83.5	38.0
Creatine	2.4	24.8	39.2^a,b^	25.8	17.7	38.2	26.0
Choline	63.4^a,b^	13.8	72.2^a,b^	32.6	46.6	29.3	60.8
PC	35.0^a,b^	25.8	30.4	33.5	25.2	54.6	55.2
Scyllo-inositol	48.1	-3.3	-63.7^a,b^	84.8	85.7	54.8	84.1
Taurine	14.4^b^	24.6	44.7^a,b^	33.9	16.3	55.8	25.8
Glycine	33.4^a,b^	60.9^a,b^	81.2^a,b^	25.1	32.3	44.5	33.1
Myo-inositol	10.7	-10.8	-49.1 ^a,b^	28.3	21.7	46.8	55.9
Lactate	71.9^a,b^	100.2^a,b^	187.8^a,b^	51.5	18.9	34.9	50.1
β-glucose	76.0^a,b^	162.0^a,b^	48.0	103.8	47.4	59.8	44.1
Histidine	34.7^a,b^	11.4	81.4^a,b^	41.8	32.8	41.5	38.6
Tyrosine	31.5^a,b^	75.7^a,b^	87.8^a,b^	39.8	29.2	62.8	32.4
Phenylalanine	24.8^a,b^	63.6^a,b^	99.1^a,b^	39.1	31.3	70.4	36.4
Uracil	35.8	58.2	72.3^a,b^	77.9	62.7	82.1	58.0
Hypoxanthine	41.1^a,b^	47.5^a,b^	49.4^a,b^	58.7	22.5	39.8	39.4
Formate	48.9^a,b^	-1.3	21.9	78.3	50.5	58.9	88.0

Percent difference was calculated from the mean values of the relative signal integrals in each group. Positive values indicate a relatively higher metabolite concentration present in the pathological tissue (NN, FA and TC) compared to the healthy control. RSD: Relative Standard Deviation; NAC: *N*-acetylated compounds; PC: phosphocholine.

^a^ *P*<0.05 using Student's t test

b *P*<0.05 using the Mann-Whitney-Wilcoxon test

 Seventeen more metabolites were found to be different in at least one comparison, e.g., NN vs. H. The highest number of significantly affected metabolites was observed for TC vs. H. Interestingly, some metabolites were deregulated only in benign tissues: 3-hydroxybutyrate and β-glucose in NN and FA, while phosphocholine and formate were only changed in NN.

 Finally, four metabolites: creatine, scyllo-inositol, myo-inositol and uracil, were found to be selective biomarker candidates for TC. 

### Pair-wise OPLS-DA for discrimination between different thyroid lesions

**Figure 3:**
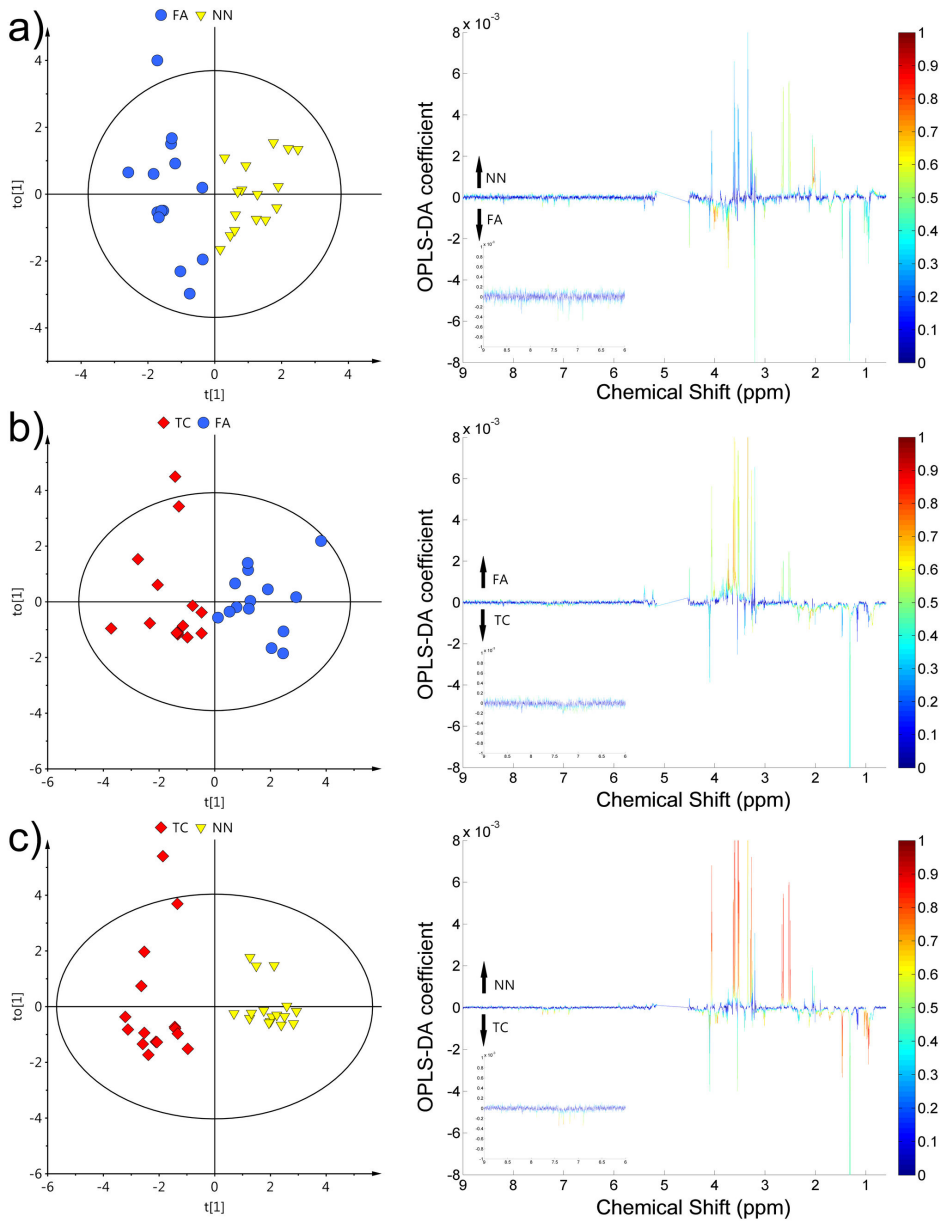
OPLS-DA results and corresponding loadings of discrimination between different thyroid lesion groups: (a) TC vs. **NN, (b) FA vs. NN, (c) TC vs. FA**. Non-neoplastic nodules - NN, follicular adenomas - FA, thyroid cancer - TC. The color bar corresponds to the absolute value of the correlation loading in the discrimination model.

 To determine if the FA group is more similar to benign or malignant tissue, a pair-wise OPLS-DA analysis between all pathological groups was conducted: FA vs. NN, FA vs. TC and TC vs. NN (Figure 3). All the obtained models were characterized by *Q* ^*2*^ *Y* values higher than 0.3. The analysis using OPLS-DA models (all built with one predictive and one orthogonal component) produced the following results: for FA vs. NN, R^*2*^ *X* = 0.242, *R* ^*2*^ *Y* = 0.795 and *Q* ^2^ *Y* = 0.368, for FA vs. TC, *R* ^*2*^ *X* = 0.301, *R* ^*2*^ *Y* = 0.745 and *Q* ^2^ *Y* = 0.442. The greatest differences were observed for TC vs. NN where *Q* ^*2*^ *Y* value crossed 0.8 resulting in a very good prediction ability, *R* ^*2*^ *X* = 0.338, *R* ^*2*^ *Y* = 0.910 and *Q* ^2^ *Y* = 0.819. 

 It was possible to discriminate between two benign thyroid lesion types. In this case, the main differences observed in the loadings plot were related to an increase of branched chain amino acids (BCAA) isoleucine and valine and decreased citrate and N-acetylated compounds (NAC) in FA compared with NN (Figure 3a). 

 The differences between FA and TC mainly involved higher scyllo-inositol and *myo*-inositol together with lower lactate and methionine concentration in FA compared to TC. BCAAs did not contribute strongly to the model (Figure 3b). 

 Finally, in the comparison of TC vs. NN, the loadings plot was a combination of the previous two models with the sum of change and involved BCAAs, lactate, scyllo-inositol, *myo*-inositol, citrate and NACs (Figure 3c). The metabolites that were determined to contribute most to class separation were next tested with univariate statistics (Table 4).

**Table 4:** Statistically important metabolites that contribute to the subsequent separation within thyroid lesion groups.

**Metabolite**	**% difference**			***P*-Value** ^c^
	**FA vs. NN**	**FA vs. TC**	**TC vs. NN**	
Isoleucine	69.4^a,b^	1.7	66.6^a,b^	0.0009
Valine	87.4^a,b^	-11.4	108.8^a,b^	0.0001
Alanine	52.6^a,b^	-24.7^b^	90.3^a,b^	0.0001
NAC	-33.5^a,b^	-7.0	-28.8^a,b^	0.0002
Methionine	3.9	-32.6^a,b^	37.8^a,b^	0.0033
Citrate	-55.4^a,b^	56.5^a,b^	-80.6^a,b^	<0.0001
Creatine	21.9	-11.6	36.0^a,b^	0.0070
Choline	-30.4^a^	-51.^b^	5.4	0.0465
GPC	16.9	25.8^a,b^	-13.3	0.0518
Scyllo-inositol	-34.7	62.4^a,b^	-75.5^a,b^	0.0005
Taurine	9.0	-16.1	26.5^a,b^	0.0723
Glycine	20.6	-12.6	35.9^a,b^	0.0450
Myo-inositol	-19.4^a^	42.9^a,b^	-54.0^a,b^	<0.0001
Lactate	16.5	-43.7^a,b^	67.4^a,b^	0.0067
Histidine	-17.3	-62.8^a,b^	34.6^a,b^	0.0069
Tyrosine	33.6	-6.9	42.8^a,b^	0.0362
Phenylalanine	31.1	-21.7^b^	59.5^a,b^	0.0050

Percent difference was calculated from mean values of relative signal integrals in each group. Positive values indicate a relatively higher metabolite concentration present in the pathological tissue calculated from left to right, e.g., the positive value in FA vs. NN means that FA is higher compared to NN. RSD: Relative Standard Deviation; NAC: *N*-acetylated compounds; GPC: glycerophosphocholine.

a *P*<0.05 using Student's t test

b *P*<0.05 using the Mann-Whitney-Wilcoxon test

c Kruskal-Wallis ANOVA

**Figure 4:**
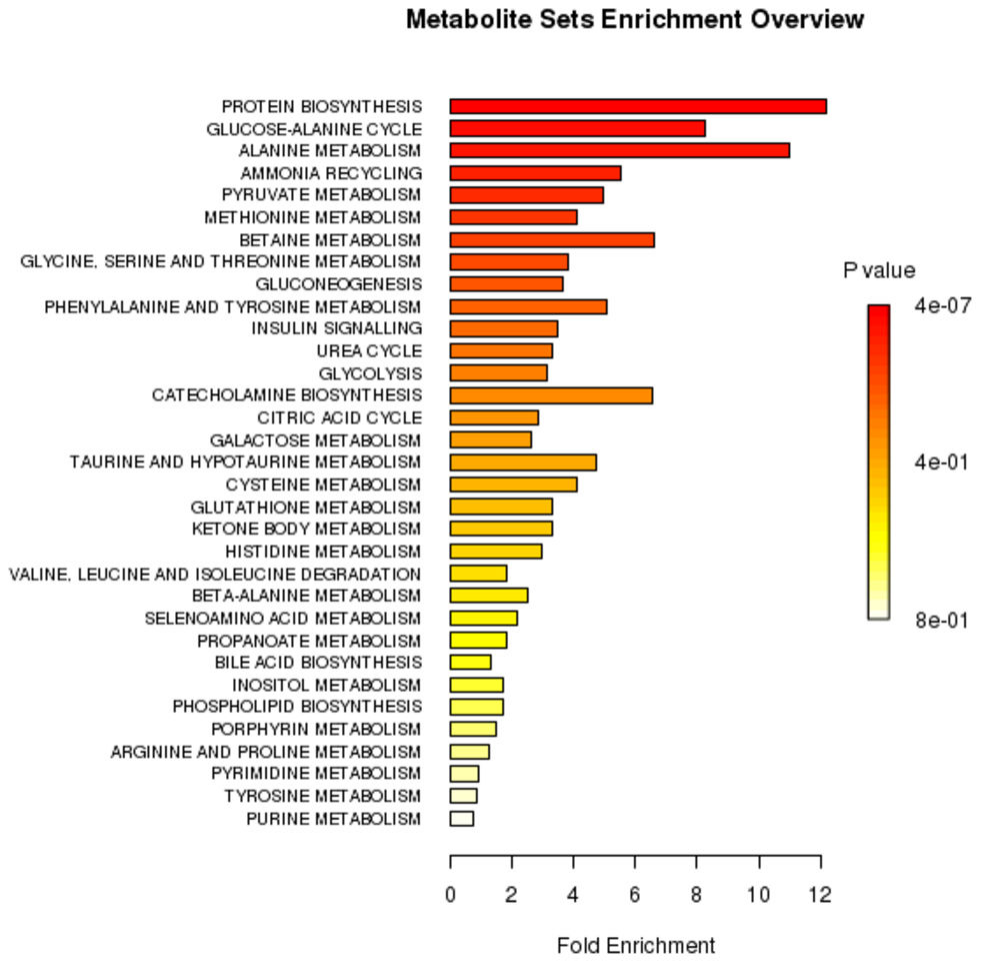
Results of metabolite set enrichment analysis (MSEA). The horizontal bar graph summarizes metabolic pathways that were strongest affected in the thyroid lesions compared to healthy thyroid.

 Metabolite set enrichment analysis (MSEA) was utilized to indicate which metabolic pathway may be the most affected by thyroid tumor development. Therefore, all metabolites that were significantly different between thyroid lesions and healthy thyroid were used for MSEA. MSEA identified the following metabolic pathways: protein biosynthesis, the glucose-alanine cycle, alanine metabolism, ammonia recycling, and pyruvate, methionine and betaine metabolism (Figure 4).

## Discussion

### Metabolic changes in thyroid lesions

 The development of cancer requires the existence of particular conditions under which cell metabolism is reprogrammed to satisfy its bioenergetic and biosynthetic requirements and to allow uncontrolled proliferation of the cells [37]. As a result, cancer tissue demonstrates a significantly different profile of low molecular weight metabolites. Diagnostically useful metabolites can be characterized in two ways: the first category are zero-one discriminators between healthy tissue and all types of lesions, while the second is metabolites which exhibit continuous changes of concentration correlating with various stages of tumor progression. 

 Based on MSEA the main differences of metabolic thyroid aqueous tissues extract profiles observed by NMR were related to protein biosynthesis. This result is not surprising because the majority of metabolic changes in living systems are the consequence of enzymatic/protein activity. The increased proliferation rate of cancer cells require fast supplementation of protein machinery. Therefore, most of the changes in amino acids levels in thyroid lesions are most likely an indication of protein anabolism and/or catabolism. 

 Our study showed that a decreased level of lipids in comparison to healthy thyroid tissue (H) is a common feature observed in all analyzed types of thyroid lesions. We used an aqueous extraction procedure in which lipid extraction efficiency could be low and not uniform across all types of analyzed samples. However, the general trend is in accordance with the available literature [29,30,]. This phenomenon is most likely related to membrane biosynthesis during uncontrolled proliferation in lesion growth. 

**Figure 5:**
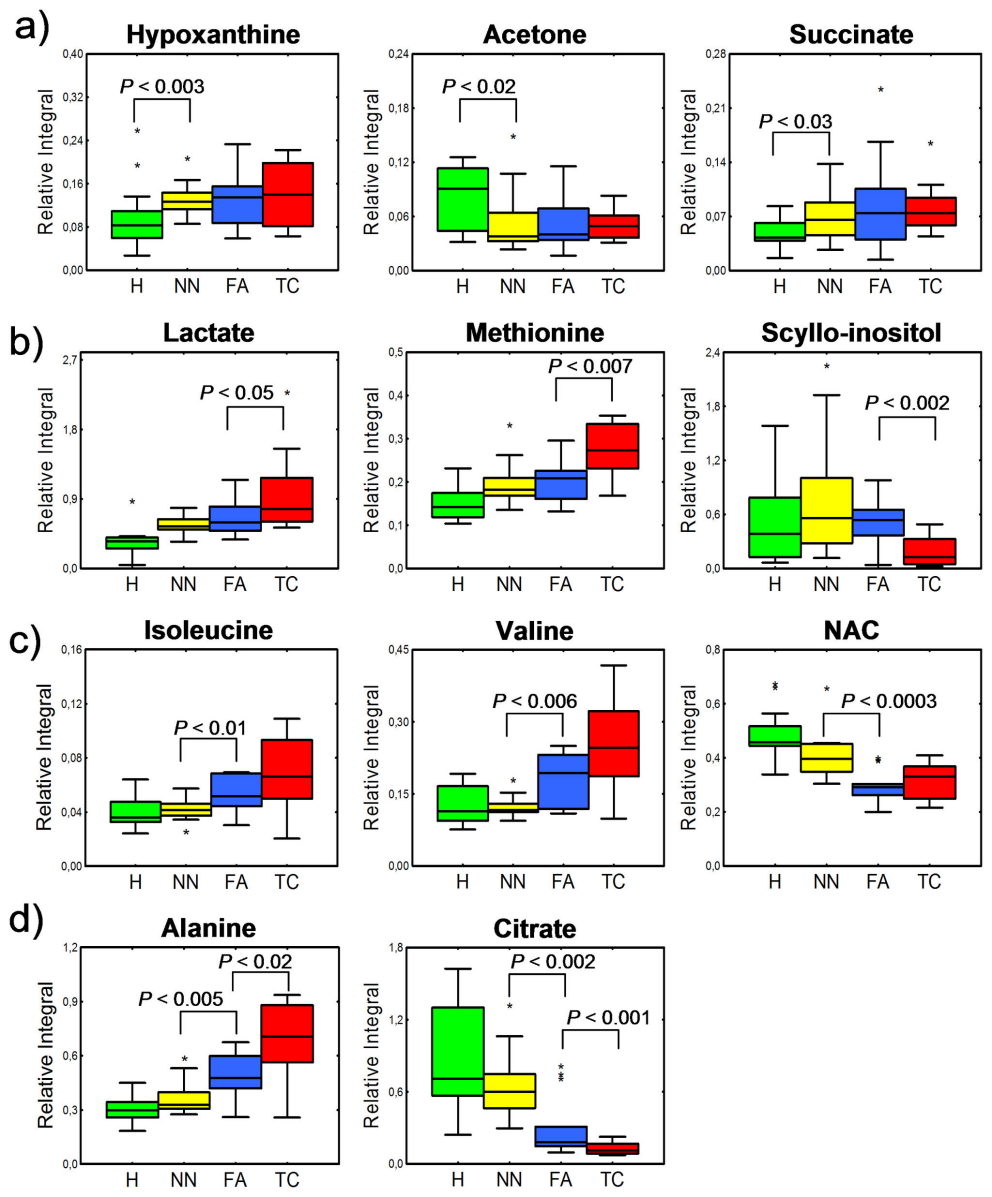
Plots representing relative integral values of selected metabolite. Healthy thyroid: H; non-neoplastic nodules: NN; follicular adenomas: FA; thyroid cancer: TC; bar: median; box: Q1-Q3 interquartile range; whiskers: non-outlier min-max range; asterisk: outlier. *P* value was obtained using the Mann-Whitney-Wilcoxon test. Metabolites are divided into four groups: (a) all thyroid lesions present different metabolite level from healthy thyroid tissue, while no significant difference can be found between thyroid lesions; (b) biomarker candidates specific to TC; (c) metabolites showing differences between two thyroid lesion groups considered as benign NN and FA. Note that FA behaves more similar to TC (malignant) than to benign NN, (d) metabolites showing intermediate character of FA.

 Interestingly, the hypoxanthine level was elevated by approximately 40 - 50% across all thyroid lesion tissues compared to healthy thyroid tissues. This result indicates a potential role in tumor development mechanisms.. Theoretically, this occurrence may be caused by the ability of certain tumor tissues to switch from a *de novo* nucleotide synthesis pathway to the more efficient salvage pathway which is reflected by increased hypoxanthine and xanthine content [38]. However, without either flux or inhibitor experiments it remains unclear whether it is due to the accumulation of this compound in tumor tissue or nucleoside breakdown. Nevertheless, while no changes of hypoxanthine were found between different thyroid lesions it can be considered as a thyroid lesion biomarker candidate. Similarly, succinate and acetone presented stable levels across thyroid lesions and were significantly different from healthy tissue (Figure 5a).

 The significantly increased levels of lactate found in thyroid cancer tissues are most likely related to the Warburg phenomenon. Instead of using pyruvate to supply TCA cycle, cancer cells overproduce lactate, which is secreted from cells. Interestingly, we observed a lactate increase not only in cancer tissues but also in benign lesion compared to healthy thyroid tissue, and this increase was statistically significant.

 The reduced levels of *scyllo*- and *myo*-inositol in thyroid cancer tissues may be related with osmoregulation of cancer cells (Figure 5b). Furthermore, the taurine concentration was increased in thyroid cancer. Taurine was previously considered as a potential indicator of tumor aggressiveness [39]. Similar to *scyllo*- and *myo*-inositol, taurine acts as an osmoregulator. The same set of affected metabolites was reported previously for lung [40], breast [41] and colon [42,43] cancers. This indicates that changes in osmoregulation are common for many cancer cells. It must be stressed that this type of dysregulation was not observed in benign tumors (NN and FA).

### Follicular adenomas exhibit a unique metabolic profile

 In our study, it was possible to clearly distinguish FA from healthy thyroid and from thyroid cancers and non-neoplastic nodules. The reasons why such discrimination could be achieved lies in the unique metabolic profile of follicular adenomas. Mountford’s group first reported nonhomogeneous behavior of FA based on lipid signals. However, in our study FA presented more metabolic changes in many metabolites in contrast to other thyroid lesions. Our results show that FA presented only some metabolites at levels of benign thyroid non-neoplastic nodules (Figure 5b). Another group of affected metabolites present in FA were at the similar level as in thyroid cancer (Figure 5c). FA exhibited increased concentrations of BCAAs and decreased NACs similar to thyroid cancer. These results may prove that some aspects of protein biosynthesis or breakdown pathways are already active in FA at the same level as in TC. Moreover, FA presents intermediate character with respect to alanine and citrate (Figure 5d). Citrate is a TCA cycle product, that was reduced significantly in all thyroid lesions. Such a phenomenon could be explained by an increased conversion of citrate into acetyl-CoA and further fatty acid biosynthesis. However, a clear trend in citrate reduction correlated with the type of thyroid lesion was observed. This trend shows intermediate character of FA that does not behave exactly like benign lesions. 

 In conclusion, FA possess metabolic features of benign thyroid lesions like those classified as NN, while simultaneously exhibiting part of a metabolic profile associated with TC. These observations may indicate an intermediate nature of FA and can be useful in further investigation of possible transition of FA into different forms of thyroid cancer.

### Role of follicular adenomas in thyroid cancer progression

 It remains unclear whether follicular thyroid carcinoma arises from preexisting follicular adenoma (FA). However, there is some evidence confirming that theory (epidemiological and demographic data, cases of malignancies found within benign nodules, rare occurrence of follicular “microcarcinoma”, coexistence of some molecular tumor markers and the identical cytological appearance) [44,45]. However, thyroid follicular adenomas and thyroid cancers show fundamentally distinct gene expression patterns. For example, it was found that more than one hundred genes differentiate follicular adenomas from carcinomas [46]. The prevailing opinion is that follicular thyroid carcinomas (FTC) develop directly from follicular cells and from benign thyroid adenomas (malignant transformation), while papillary thyroid carcinomas (PTC) originate exclusively from follicular cells. Additionally poorly differentiated or anaplastic carcinomas can arise from FTC and PTC or develop *de novo* [1]. However, there is also research demonstrating the possibility that one subset of follicular adenomas could progress to the follicular carcinoma stage, while the second subset of follicular adenomas could progress to papillary carcinomas [47].

## Conclusion

 To the best of our knowledge, this is the first study focused on NMR analysis of aqueous thyroid tissue extracts. The approach is alternative to typical methods, such as tissue extraction using organic solvents or HR MAS NMR analysis of intact tissue. We are aware that metabolic profile of aqueous tissue extract may be altered from the original *in vivo* state. However, our study was performed carefully and the observed changes are mostly in agreement with literature data [24,29,30]. Additionally, our study correctly discriminated between the analyzed groups. Nevertheless, all samples were prepared in the same protocol and reflects systematical changes occurring in each type of analyzed thyroid lesion.

 Based on the combined metabolomics analyses of three types of thyroid lesions and healthy thyroid tissue, characteristic ^1^H NMR profiles were assessed and potential biomarkers common to all thyroid lesions were identified, namely: alanine, methionine, acetone, glutamate, glycine, lactate, tyrosine, phenylalanine and hypoxanthine. Moreover, *scyllo*- and *myo*-inositol were found to be specific biomarkers of thyroid cancer. This study demonstrates the utilization of the NMR-based analysis of thyroid aqueous tissue extract combined with chemometric modeling and explores its potential as an auxiliary diagnostic tool of thyroid lesions. According to histopathological classification, both non-neoplastic nodules - (NN) and follicular adenomas (FA) are considered as benign. However, according to this study FA exhibits a unique metabolite composition, that allows for discrimination from NN with almost the same accuracy as from TC. No similar significant differences have been presented in the available literature. Our findings indicate that FA could be an individual stage of carcinogenesis and suggests FA is between NN and TC. 

 This demonstrated method can be used as a complementary tool for studying cancer tissue metabolic profiles.
